# Fisetin ameliorates oxidative glutamate testicular toxicity in rats via central and peripheral mechanisms involving SIRT1 activation

**DOI:** 10.1080/13510002.2022.2116551

**Published:** 2022-09-01

**Authors:** Fatma H. Rizk, Nema A. Soliman, Suzan E. Abo-Elnasr, Heba A. Mahmoud, Muhammad T. Abdel Ghafar, Rasha A. Elkholy, Ola A. ELshora, Reham A. Mariah, Shaimaa Samir Amin Mashal, Amira A. El Saadany

**Affiliations:** a Department of Medical Physiology, Faculty of Medicine, Tanta University, Tanta, Egypt; b Department of Medical Biochemistry, Faculty of Medicine, Tanta University, Tanta, Egypt; c Department of Histology and Cell Biology, Faculty of Medicine, Tanta University, Tanta, Egypt; d Department of Pharmacology, Faculty of Medicine, Tanta University, Tanta, Egypt; e Department of Clinical Pathology, Faculty of Medicine, Tanta University, Tanta, Egypt; f Department of Internal Medicine, Faculty of Medicine, Tanta University, Tanta, Egypt

**Keywords:** Monosodium glutamate, oxidative stress, fisetin, testicular toxicity, SIRT1, NOX4, reduced glutathione, hypothalamic-pituitary-gonadal axis

## Abstract

**Objectives:**

This study aimed to evaluate the potential mitigating effect of fisetin on monosodium glutamate (MSG)-induced testicular toxicity and investigate the possible involvement of silent mating type information regulation 2 homolog 1 (SIRT1) in this effect.

**Methods:**

Forty male rats were divided into normal control, fisetin-treated, MSG-treated, and fisetin + MSG-treated groups. Testosterone, GnRH, FSH, and LH were measured in plasma, as well as SIRT1 and phosphorylated AMP-activated protein kinase (pAMPK) levels in testicular tissues using ELISA. Hydrogen peroxide (H_2_O_2_), nitric oxide (NO), and reduced glutathione (GSH) were measured colorimetrically, while *Nicotinamide adenine dinucleotide phosphate oxidase 4 (NOX4)* expression was relatively quantified using RT–PCR in testicular tissues.

**Results:**

After 30 days, fisetin could ameliorate MSG-induced testicular toxicity by acting centrally on the hypothalamic-pituitary-gonadal axis, increasing plasma levels of GnRH, FSH, LH, and testosterone. Peripheral actions of fisetin on the testis were indicated as it increased testicular SIRT1 and pAMPK. Furthermore, it antagonized glutamate-induced oxidative stress by significantly lowering H_2_O_2_, NO, and relative *NOX4* expression while significantly increasing reduced GSH levels. It also improved the architecture of the seminiferous tubules, reduced sperm abnormality, and increased sperm count.

**Discussion:**

Fisetin ameliorates MSG-induced testicular toxicity via central and peripheral mechanisms making it a promising therapeutic target for male infertility.

## 1. Introduction

Currently, there is a worldwide concern about increased male infertility, which is influenced by various risk factors such as exposure to hazardous chemicals, environmental factors, nutritional factors, weight, physical activity, stress, smoking, alcohol use, medications, diabetes, clothing, and sleep. These factors mainly affect spermatogenesis and sperm quality [1].

Monosodium glutamate (MSG) is a synthetic sodium salt of the non-essential amino acid glutamate. It is a flavor-enhancing food additive that is widely used in many food ingredients, mostly without labeling. Thus, the amount of MSG consumed daily by the average person cannot be accurately calculated [2]. After oral administration, it is absorbed from the gastrointestinal tract and raises the plasma level of glutamate, which binds to glutamate receptors in the testis, resulting in oxidative glutamate toxicity, testicular hemorrhage [3], oligospermia, and sperm abnormality in rats [4].

Fisetin (3,7,3′,4′-tetrahydroxyflavone) is a bioactive phytochemical found in a variety of fruits and vegetables, including strawberries, apples, onions, and cucumbers. It has been suggested that fisetin acts as a potent antioxidant that protects the brain from oxidative glutamate toxicity [5]. It can cross the blood-testis barrier, as fisetin has been shown to improve the efficacy of treatment of testicular carcinoma *in vivo* [6]. Therefore, fisetin may be a promising therapy for oxidative glutamate testicular toxicity.

The silent information regulator of transcription 1 (SIRT1) is a NAD-dependent deacetylase protein. It regulates many cellular processes in the sperm, including energy homeostasis via adenosine monophosphate-activated protein kinase (AMPK) activation [7], DNA repair, autophagy, cell survival, and apoptosis [8]. To our knowledge, no reports exist on the effects of MSG and fisetin on testicular SIRT1 and its downstream signaling pathways. In addition, previous reports have indicated that activating SIRT1 could be a therapeutic target for male infertility [9]. Therefore, the current study aimed to investigate the potential mitigating effect of fisetin on MSG-induced testicular toxicity as well as the possible role of SIRT1 in this effect.

## 2. Material and methods

### 2.1. Drugs and chemicals

Fisetin was supplied as a crystalline powder (Naturewill Biotechnology Co., Ltd., China) and was dissolved in dimethyl sulfoxide (DMSO) and then in phosphate-buffered saline (PBS, pH 7.2) in a 1:1 solution of DMSO: PBS. MSG was supplied in the form of crystalline powder and was prepared by dissolving the calculated dose in 0.5 ml of distilled water, according to the animal weight. Unless otherwise specified, all chemicals and solvents used in this experiment were purchased from Sigma (Sigma Chemicals Co., St. Louis, MO, USA) and were of high analytical grade.

### 2.2. Animals

In this experiment, 40 male Wistar albino rats weighing 180–200 g were used. The study was conducted in March 2021 at the Department of Pharmacology, Faculty of Medicine, Tanta University. The animals were randomly distributed to well-ventilated cages with five rats each, under strict hygienic measures, at room temperature 25 ± 2°C, and with free access to food and water. Two weeks prior to the start of the experiment, the rats were kept for acclimatization. All experiments were done at the same time of day. This study followed the National Institutes of Health guidelines for the care and use of laboratory animals (NIH Publications No. 8023, revised 1978). The experimental protocol was approved by the animal care review committee at the Faculty of Medicine, Tanta University, Egypt (approval code NO.34586/3/21) and was in accordance with the ARRIVE guidelines.

The rats were randomly divided into four groups of ten each, and they were given the following treatment for 30 days: Group I (normal control group) received DMSO and PBS (1:1 solution) via intraperitoneal (i.p) injection twice a week and distilled water via oral gavage daily in the same doses as groups II and III. Group II (fisetin group) received 20 mg/kg fisetin via i.p. injection twice a week. This dose was chosen based on previous research findings that fisetin administration at this dose could significantly reduce oxidative stress and systemic inflammation in an adult mouse model of memory dysfunction [10], and improve metabolic dysfunctions in nonalcoholic fatty liver disease in mice via activation of the sirt1/AMPK and β-oxidation pathways [11]. Group III (MSG group) received MSG (1 g/kg/day) by oral gavage daily, as used in previous studies to simulate the average amount of MSG consumed by humans as a food additive [12]. Group IV (the fisetin + MSG group) received both fisetin and MSG concomitantly, as did groups II and III.

### 2.3. Blood and tissue collection and preparations

By the end of the experiment, the rats had been anesthetized with an i.p. injection of sodium pentobarbital (60 mg/kg) [13]. Blood samples were drawn via a cardiac puncture, collected in tubes containing heparin for plasma separation, and stored at −80°C until biochemical analysis of sex hormones. The abdominal cavity was opened to expose the testis, which was quickly dissected out, washed with ice-cold saline, cut into multiple small pieces, and weighed. All specimens included interstitial areas and seminiferous tubules. One piece was taken and immediately fixed in Bouin’s solution for 24 h, then dehydrated in ascending grades of ethanol, cleared, and embedded in paraffin. Serial sections of 5 μm thickness were stained with hematoxylin and eosin for routine histopathological examination [14]. The second one was homogenized with 50 mM PBS (pH 7.4) on ice, and Phosphatase and Protease Inhibitor Cocktails (Sigma-Aldrich, Missouri, United States) were added to samples according to the manufacturer’s instructions. The supernatant was separated by centrifugation at 9000 rpm for 20 min at 4°C, and the resultant supernatant (free of insoluble materials) was frozen at −80°C until analysis. The residual testicular tissue was stored in liquid nitrogen at −80°C for RNA extraction. Tissue protein content was determined by the method of Lowry et al. [15].

The epididymis was used to prepare sperm to study sperm morphology. An equal piece of the cauda epididymis from each rat was cut out, incised by making small cuts, and placed in 4 ml of saline to allow sperm to swim out. After 20 min, the cauda epididymis was removed, and the suspension was gently shaken to homogenize [16]. Following that, the samples were stained with eosin to make it easier to visualize the sperm. In a test tube, 1 mL of sperm suspension was placed. Two drops of 1% (w/v) eosin Y were added to the test tube and gently mixed. To allow for staining, sperm was incubated at room temperature for approximately 45–60 min and then resuspended with a Pasteur pipette [17]. One to two drops of the stained sperm suspension were placed about 1 cm from the frosted end of a pre-cleaned microscope slide and gently spread out. The slides were examined at 1000× using a Leica light microscope to look for any abnormalities in the morphology of the sperms. At least five sequential fields were examined. On a single slide, up to 200 sperms were counted [18]. Normal and abnormal sperm were differentiated. The abnormal sperms had either head or tail or head and tail abnormalities. The sperm abnormality results were expressed as a percentage of normal sperm [19].

The sperm was counted using a hemocytometer. To load the sample into the hemocytometer’s chamber, 0.9 ml of PBS was added to 0.1 ml of the prepared semen sample, gently mixed, and 10 µL of the prepared sample was placed in the V-shaped groove of the hemocytometer by the tip of the micropipette [20]. The sample was allowed to settle for 2–3 min. The sperm number was counted in the hemocytometer’s central counting area, which has 25 large squares and 16 smaller squares within each large square. All sperm in the 25 large squares were counted and the number of sperm per ml was calculated as the mean number of sperm counted in each chamber × 10,000 × dilution factor [21].

### 2.4. Assessment of plasma hormones

Plasma levels of testosterone, follicle-stimulating hormone (FSH), and luteinizing hormone (LH) were measured using an enzyme-linked immunosorbent assay (ELISA) technique with commercially available kits (Cloud-Clone Corp., Houston, USA), while plasma gonadotropin-releasing hormone (GnRH) was measured using commercially available ELISA kits supplied by LifeSpan BioSciences, Inc., Washington, USA.

### 2.5. Immunoassay

SIRT1 and phosphorylated AMPK (pAMPK) levels in the testicular tissues were measured using commercial ELISA kits (Sunred Biological Technology Co., Ltd. Shanghai, China; Glory science Co., USA, respectively), according to the manufacturer’s guidelines.

### 2.6. Assessment of oxidant/antioxidant status in testicular tissues homogenate

The levels of hydrogen peroxide (H_2_O_2_), nitric oxide (NO), and reduced glutathione (GSH) were measured colorimetrically on a Biosystem semiautomatic analyzer spectrophotometer (BTS 350, Spain) using commercial kits supplied by Biodiagnostic, Giza, Egypt.

### 2.7. Relative gene expression of Nicotinamide adenine dinucleotide phosphate oxidase 4 (NOX4)

Frozen testicular tissues were processed, and total RNA was extracted using the Qiagen RNeasy total RNA isolation kit (Qiagen, Hiden, Germany) according to the manufacturer’s instructions. cDNA was synthesized using the SuperScript® III First Strand cDNA synthesis kit (Thermo Fisher Scientific, Waltham, Massachusetts, USA). PCR reactions were performed using Power SYBR Green PCR Master Mix (Thermo Fisher Scientific, Waltham, Massachusetts, USA). *NOX4* mRNA expression was measured in relation to the housekeeping gene, *glyceraldehyde 3-phosphate dehydrogenase (GAPDH)*. Primers were designed with Primer3 software (http://bioinfo.ut.ee/primer3/) as shown in Table 1. The cycling condition was as follows: initial denaturation (95°C for 10 min), followed by 40 cycles of 95°C for 15 s, 65°C for 1 min, and 72°C for 1 min. Relative gene expression was automatically calculated from the comparative cycle threshold values of the target and the reference gene by RotorGene Q6plex and its specific software (Qiagen, Valencia, CA, USA).

**Table 1.** Primers’ sequences of NOX4 and reference gene used in reverse transcription-polymerase chain reaction.

Gene	Forward (5′–3′)	Reverse (5′–3′)	GenBank Accession No.
*NOX4*	5′-GGATCACAGAAGGTCCCTAGC-3′	5′- AGAAGTTCAGGGCGTTCACC-3′	NM_053524.1
*GAPDH*	5′-GGTGAAGTTCGGAGTCAACGGA-3′	5′-GAGGGATCTCGCTCCTGGAAGA-3′	NM_017008

Note: GAPDH, Glyceraldehyde 3-phosphate dehydrogenase; NOX4, Nicotinamide adenine dinucleotide phosphate oxidase 4.

### 2.8. Statistical analysis

Data were analyzed using Statistical Package for the Social Sciences (SPSS) software version 23.0 (IBM Corp, Armonk, NY, USA). Data were assessed for normality of distribution using the Shapiro-Wilk’s test. All data were found to be normally distributed, presented as mean and standard deviation (SD), and compared between the studied groups using one-way analysis of variance (ANOVA) followed by a post-hoc test (Tukey’s test) for multiple comparisons between different groups. *P*-values of less than 0.05 were considered statistically significant.

## 3. Results

### 3.1. Effect of fisetin and MSG on hypothalamic-pituitary-gonadal axis

**Figure 1.**
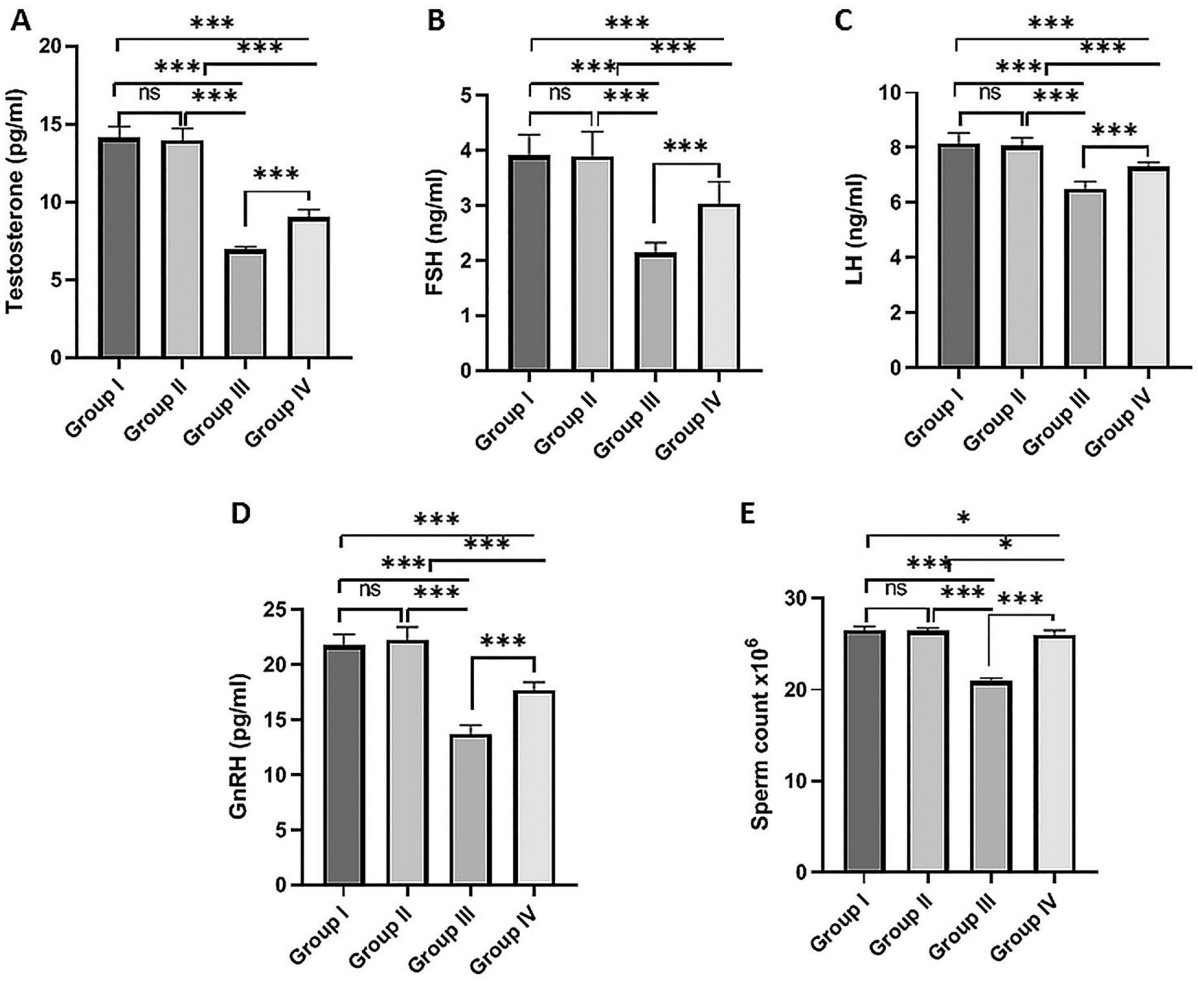
Effect of fisetin and MSG on plasma sex hormones and sperm count. (A) Testosterone, (B) FSH, (C) LH, (D) GnRH, and (E) sperm count. Note: All data are normally distributed and are expressed as mean and standard deviation. ns: non-significant, *p < 0.05, **p < 0.01, and ***p < 0.001. FSH, Follicle-Stimulating Hormone; LH, Lutenizing Hormone; GnRH, Gonadotropin-releasing hormone.

The results revealed that MSG-treated rats had significantly lower plasma testosterone (Figure 1A), FSH (Figure 1B), LH (Figure 1C), GnRH (Figure 1D) levels than the normal control group. On the other hand, concomitant administration of fisetin with MSG significantly increased these hormones compared to the MSG-treated group. In addition, the fisetin group revealed no significant changes in these hormone levels when compared to the normal control group (Figure 1).

### 3.2. Effect of fisetin and MSG on sperm count

The sperm count was significantly lower in the MSG-treated group than in the normal control group. However, co-administration of fisetin with MSG significantly increased sperm count compared to the MSG-treated group. No significant changes were detected in sperm count between the fisetin-treated group and the normal control group (Figure 1E).

### 3.3. Effect of fisetin and MSG on SIRT1 and pAMPK in testicular tissues

**Figure 2.**
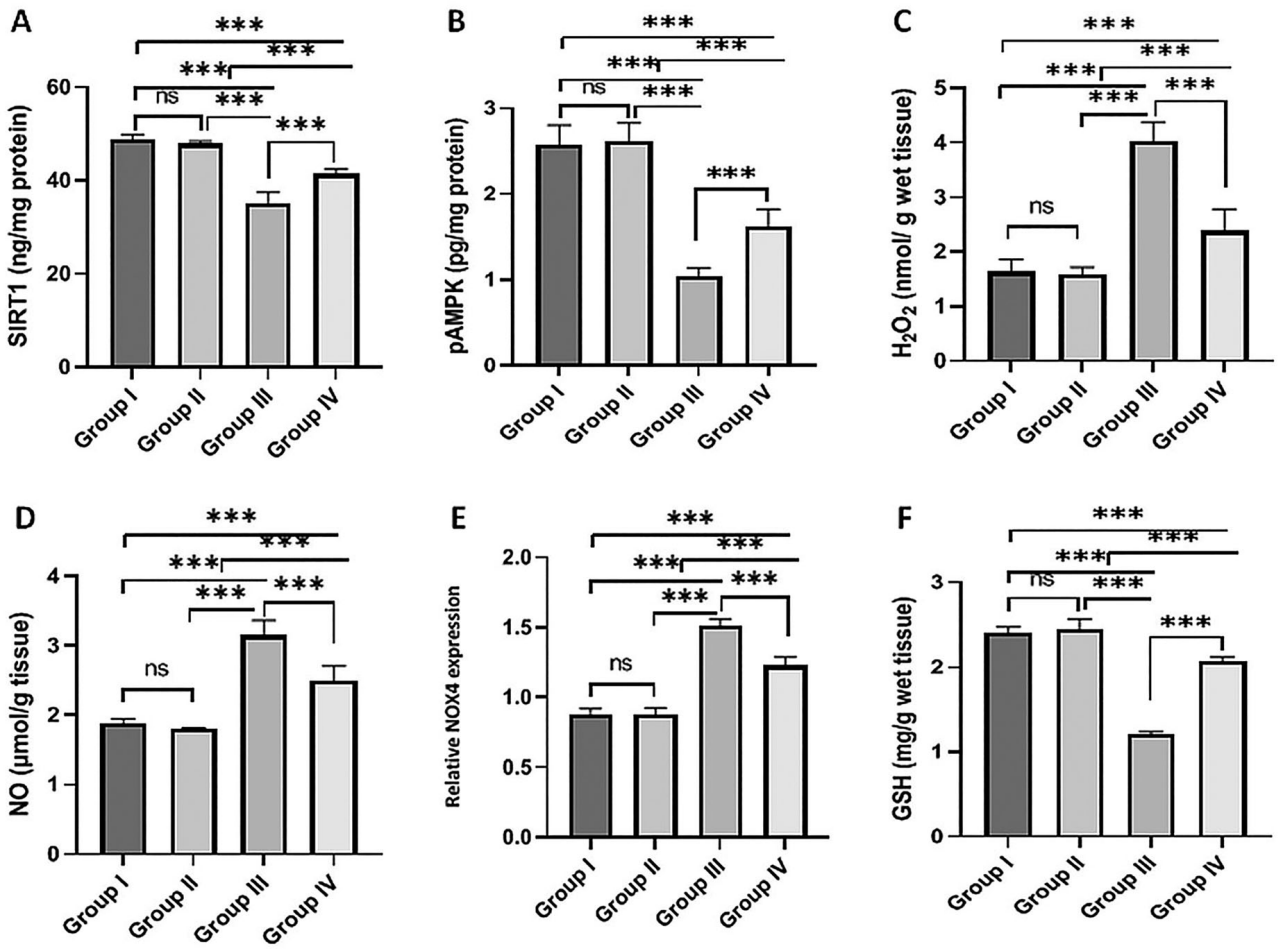
Effect of fisetin and MSG on testicular tissues markers. (A) SIRT1, (B) pAMPK, (C) H_2_O_2_, (D) NO, (E) relative *NOX4* expression, and (F) GSH. Note: All data are normally distributed and are expressed as mean and standard deviation. ns: non-significant, *p < 0.05, **p < 0.01, and ***p < 0.001. SIRT1, silent mating type information regulation 2 homolog1; pAMPK, phosphorylated AMP-activated protein kinase; H_2_O_2_, Hydrogen peroxide; NO, Nitric oxide; GSH, Reduced glutathione; NOX4, nicotinamide adenine dinucleotide phosphate oxidase 4.

Testicular SIRT1 and pAMPK levels were significantly decreased in the MSG-treated group compared to the normal control group. Meanwhile, rats treated with MSG co-administered with fisetin showed significantly higher SIRT1 and pAMPK levels than the MSG-treated group. SIRT1 and pAMPK levels in the fisetin-treated group were not significantly different from those in the normal control group (Figure 2A and B).

### 3.4. Effect of fisetin and MSG on oxidant/antioxidant status in testicular tissues

MSG administration for 30 days increased H_2_O_2_ (Figure 2C) and NO (Figure 2D) levels, and relative *NOX4* expression (Figure 2E) significantly when compared to the normal control group. However, co-administration of fisetin with MSG significantly reduced H_2_O_2_ and NO levels, as well as relative *NOX4* expression, when compared to the MSG-treated group. GSH levels decreased significantly in the MSG-treated group compared to the normal control group. However, concomitant administration of fisetin and MSG resulted in significant increases in GSH levels compared to the MSG-treated group (Figure 2F). Fisetin administration had no significant effect on H_2_O_2_, NO, GSH levels, or relative *NOX4* expression when compared to the normal control group (Figure 2).

### 3.5. Effect of fisetin and MSG on the histological structure of testis

**Figure 3.**
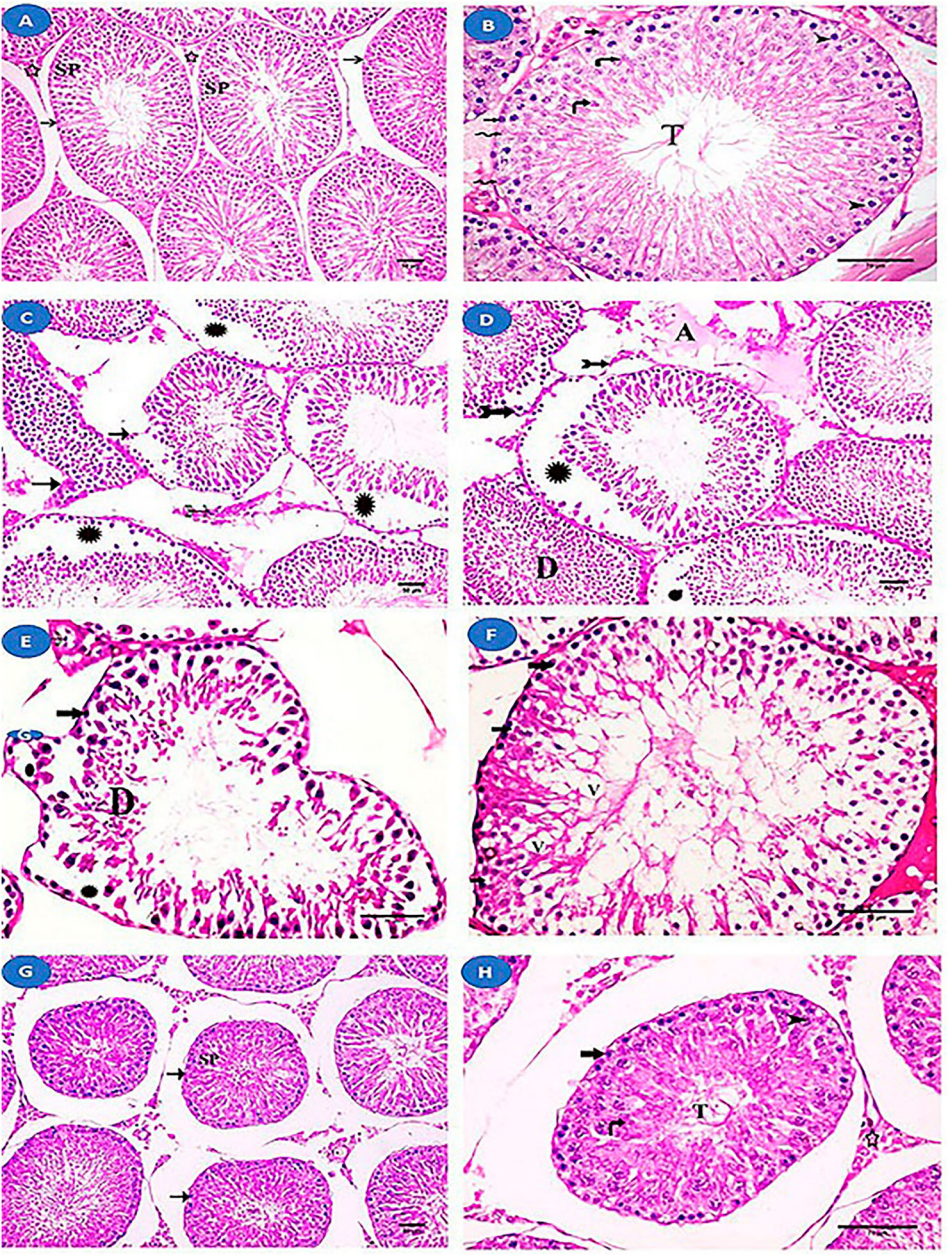
Histological examination of the testis from the studied groups. (A, B) Normal control and fisetin groups with normal seminiferous tubules (→) lined with spermatogenic cells (SP) and Sertoli cells (zigzag arrow). Interstitial tissue (a star) between the tubules can be seen. The layers of spermatogenic cells are observed. Spermatogonia (thick arrow), primary spermatocyte (arrowhead), rounded spermatids (curved arrow), and sperm tail (T) can all be seen. (C-F) The MSG-treated group showing an abnormal increase in the spaces between the seminiferous tubules with irregular outlines (→) and focal separation of their basement membrane (bifid arrows). Wide intercellular spaces and separation (Asterix) between spermatogenic cells are observed. Homogenous acidophilic material (A) is observed between these tubules. There is an apparent decrease in the layers of spermatogenic cells and spermatogonia (thick arrow) with dark nuclei and vacuolated cytoplasm. Vacuoles (V) are seen between spermatogenic cells and desquamated cells (D) in the lumen. Leydig cells (feathered arrows) with dark nuclei and vacuolated cytoplasm. (G, H) fisetin + MSG group showing improvement in the architecture of the seminiferous tubules (→), with wide spaces between them and nearly normal spermatogenic cells (SP). Spermatogonia (thick arrow), primary spermatocyte (arrowhead), spermatid (curved arrow), sperm tail (T), and interstitial tissue (star) are nearly normal structure. Hematoxylin and eosin staining (A, C, D, G) (×200, scale bar = 50 µm) and (B, E, F & H) (×400, scale bar = 70 µm).

Histological examination of the control and fisetin groups revealed that the seminiferous tubules of the testis were normal in structure (Figure 3A and B). However, histopathological examination of the MSG group revealed a change in the structure of the seminiferous tubules, including an abnormal increase in their spacing, their irregular outlines, and focal separation of their basement membrane. Additionally, wide intercellular spaces and separation of spermatogenic cells were observed. There was an apparent decrease in the layers of spermatogenic cells, and the majority of them had dark nuclei and vacuolated cytoplasm, with desquamated cells in the lumen. Between these tubules, a homogenous acidophilic material was observed. Interstitial Leydig cells showed dark nuclei and vacuolated cytoplasm (Figure 3C–F). Meanwhile, the fisetin + MSG group demonstrated improvement in the architecture of the seminiferous tubules, but wide spaces between them and nearly normal spermatogenic cells remained (Figure 3G and H).

### 3.6. Effect of fisetin and MSG on the histological structure of sperm

**Figure 4.**
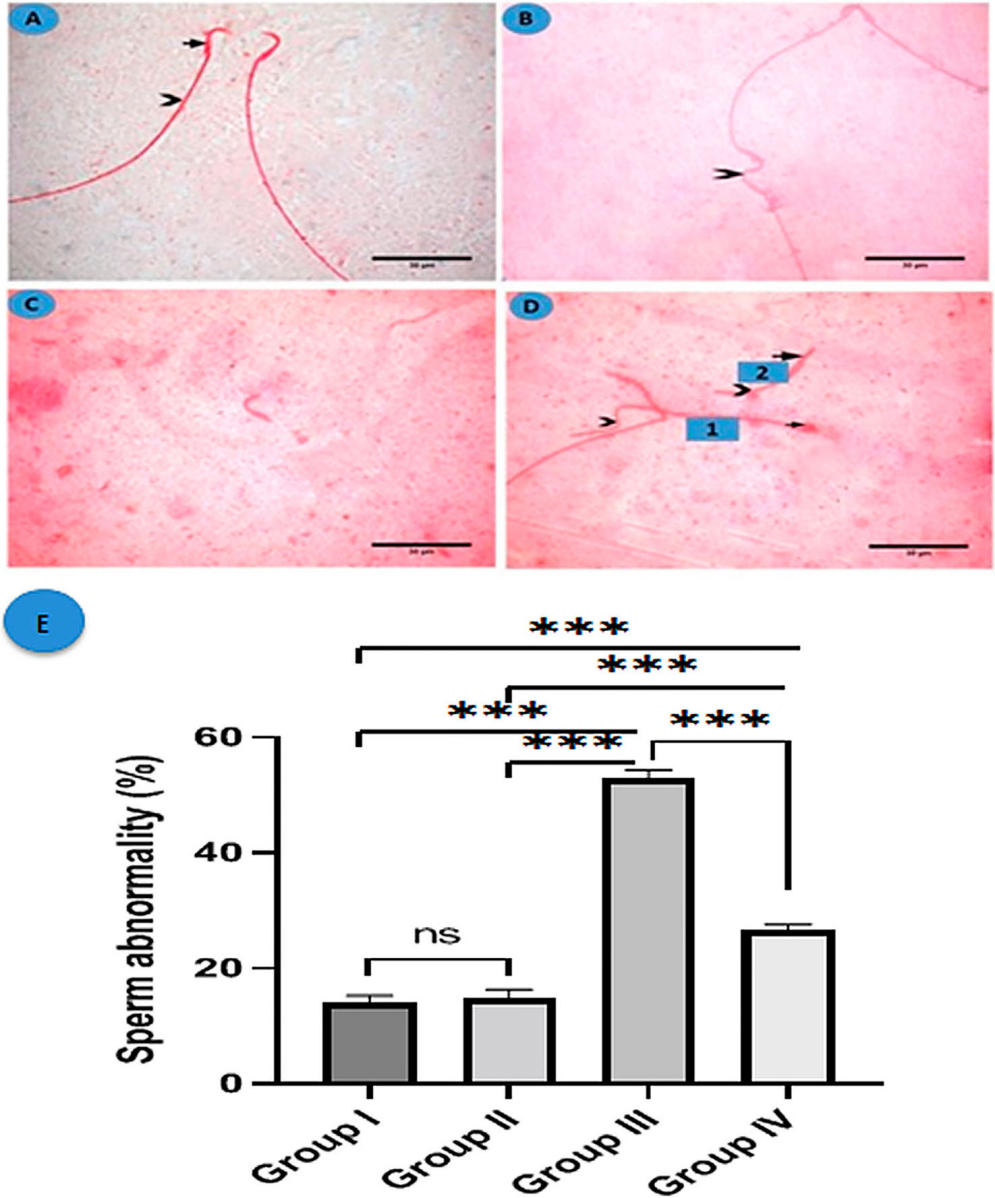
Histological structure of sperm in the studied groups. Eosin Y (A) A photomicrograph of a normal sperm from the control group shows a normal head with a characteristic hook (arrow) and a regular long tail (arrowhead). (B-D) MSG group: (B) showing angulated tail (arrowhead), (C) showing a tailless head (absent tail), and (D) showing two sperms, sperm number (1) exhibits ballooned head (arrow) with a coiled short tail (arrowhead) while sperm number (2) shows hookless head (arrow) and a short tail (arrowhead) (×1000, scale bar = 30 µm). (E) Morphometric analysis of sperm abnormalities. All data are normally distributed and are expressed as mean and standard deviation. ns: non-significant, *p < 0.05, **p < 0.01, and ***p < 0.001.

Sperm from the control and fisetin groups had a normal head with the characteristic hook and a long, regular tail (Figure 4A). However, sperm from group III had abnormal tails, including those that were absent, angulated, or short. Also, the head displayed abnormalities such as hookless and ballooned heads (Figure 4B–D). A morphometric analysis of sperm abnormalities (head or tail abnormalities) revealed a statistically significant increase in the MSG-treated group when compared to the control and fisetin groups. However, group IV revealed a significant decrease in sperm abnormality when compared to the MSG-treated group, with no significant changes between the control and fisetin groups (Figure 4E).

## 4. Discussion

The current study found that MSG administration, in a dose of (1 g/kg/day) for 30 days, induced testicular toxicity in rats, as evidenced by histopathological changes in the testis that revealed abnormalities in the structure of seminiferous tubules and spermatogenic cells, as well as morphological abnormalities of the sperm and a significant decrease in sperm count. The MSG dose used in this study was chosen to mimic as closely as possible the amount of MSG consumed by humans as a food additive. Data on dietary MSG human intake vary by region, with the average intake in the United Kingdom being 590 mg MSG per day, while extreme users consume 2330 mg MSG per day [22] and may reach 5000 mg MSG per day in highly seasoned restaurant meal intakes [23]. So previous studies used different doses of MSG to induce reproductive effects in male rats, such as Ochiogu et al. [12], who used three graded doses (0.25, 0.50, and 1.00 g/kg) and all of them resulted in a significant decrease in GnRH, LH, and testosterone levels, whereas Reynolds et al. [24] used 1, 2, 4 g of MSG to produce reproductive effects. Thus, the dose used in this study approximated an average effective dose of MSG that produces reproductive effects. However, co-administration of fisetin with MSG could antagonize MSG-induced testicular toxicity, as evidenced by improvements in the architecture of the seminiferous tubules, decreased morphological abnormalities of the sperm, and a significant increase in sperm count. Disturbed spermatogenesis could be caused by intrinsic problems with the germ cells, the supporting Sertoli cells, the Leydig cells, or changes in the endocrine signaling that supports the entire process [25]. The current study found that MSG disrupted the hypothalamic-pituitary-gonadal axis by lowering levels of GnRH, LH, FSH, and testosterone, all of which are required for normal testicular function and spermatogenesis. MSG has a central effect on the hypothalamic-pituitary-gonadal axis by excessively activating N-methyl-D-aspartic acid (NMDA) glutamate receptors in the hypothalamus, resulting in calcium influx and neuronal death via an excitotoxic pathway. This reduces GnRH in the hypothalamus with subsequent effects on the hypothalamic-pituitary-gonadal axis. The lack of LH and FSH production and release from the anterior pituitary gland reduces testosterone production by Leydig cells and disrupts spermatogenesis [26]. On the other hand, fisetin in this study was shown to improve the decreased plasma sex hormone levels caused by MSG by acting centrally on the hypothalamic-pituitary-gonadal axis.

Furthermore, MSG may induce testicular toxicity by acting peripherally on the testis. Previous studies have shown that glutamate receptors and transporters are present in the cells of the seminiferous tubules [27]. Immunohistochemical and molecular biological analyses of rat testis revealed the expression of glutamate NMDA receptor (NR1) in the germinal epithelium and interstitial spaces, GluR1 mRNA in Sertoli cells, GluR2/3 mRNA in interstitial spaces, GluR2/3 proteins in interstitial cells and arteriolar wall, and GluR5 mRNA in germinal cells except for spermatogonia [28]. The presence of glutamate receptors in the seminiferous tubules could explain the current study’s findings that MSG decreased testicular SIRT1 and pAMPK levels. On the other hand, fisetin counteracted these effects.

SIRT1 is required for normal spermatogenesis and germ cell differentiation. Mice lacking SIRT1 in male germ cells had lower sperm counts and more abnormal sperm [25]. Previous studies have indicated that SIRT1 regulates a variety of active substances. It increases AMPK phosphorylation and activation [29], which regulates many processes of cellular metabolism [30]. SIRT1 and pAMPK collaborate to activate each other, induce mitochondrial biogenesis, and serve as antioxidants [29]. SIRT1 activates peroxisome proliferator-activated receptor gamma coactivator 1 alpha (PGC1α), a master regulator of mitochondrial biogenesis and functions [31]. Furthermore, pAMPK stimulates ATP production by increasing the activity and expression of proteins involved in catabolism, which affects sperm motility and acrosomal reaction. In addition, pAMPK decreases reactive oxygen species (ROS) levels, including the H_2_O_2_ system, and increases the activity of several antioxidant enzymes [9].

In this study, MSG increased testicular ROS and nitrogen species including H_2_O_2_ and NO levels as well as relative NOX4 gene expression, while decreasing antioxidant capacity by decreasing GSH levels, proving glutamate-induced oxidative stress. These findings were consistent with Al-Shahari and El-Kott [4], who found that MSG induces oxidative stress in rats. MSG-induced oxidative stress could be explained by exogenous glutamate’s ability to prevent cystine uptake via the cystine/glutamate antiporter, resulting in intracellular GSH depletion [32]. GSH is an endogenous antioxidant protects cells from oxidative damage by that scavenging free radicals [33]. Furthermore, MSG increased NO levels by increasing the gene expression of the inducible NO synthase (iNOS) [34]. NO, a free radical, reacts with superoxide radical to form peroxynitrite, which initiates strong oxidation reactions as it interacts directly with protein and non-protein thiol groups, leading to the depletion of cellular antioxidant defences such as GSH [35].

This study introduced a novel mechanism to explain MSG-induced oxidative damage in testicular tissue by increasing *Nox4* gene expression. Nox4 is an outlier in the Nox family of NADPH oxidases because it generates H2O2, whereas Nox1, Nox3, and Nox5 generate superoxide ($O_{2}^{-}$) [36]. Thus, Nox4 is an active participant in the tissue vasculature because it induces H_2_O_2_ production, which promotes vasodilation [37]. It has been suggested that testicular vasodilatation raises testicular temperature, causing Leydig cell damage and decreasing testosterone levels [38]. Furthermore, H_2_O_2_ can rapidly kill any type of cell by producing highly reactive hydroxyl radicals that can attack cellular or organelle membranes phospholipids or polyunsaturated fatty acids, resulting in the formation of various types of aldehydes, such as malondialdehyde [39]. ROS endanger the reproductive system’s health, particularly testicular function. Attacks by free radicals can cause arterial occlusion and serious damage to reproductive system cells, which disrupts spermatogenesis [40].

Meanwhile, the current study found that fisetin has antioxidant effects, as evidenced by decreased levels of oxidants such as H_2_O_2_, NO, and *NOX4* while increasing antioxidant capacity by increasing GSH levels. Previous studies in other organs, including the hippocampus [10] and kidneys [41], have shown that fisetin has antioxidant effects. These effects may be explained by fisetin’s ability to increase GSH levels by inducing the major transcription factors that maintain GSH levels, enhancing the glutamate-cysteine ligase (GCL) activity; the rate-limiting enzyme of GSH biosynthesis [42], and preventing peroxynitrite, which depletes GSH [43]. Furthermore, fisetin inhibits iNOS expression, which regulates NO production [42], and has a high ability to donate a hydrogen atom from the hydroxyl group and scavenge free radicals [44].

In addition, fisetin activates SIRT1, which protects germ cells from apoptosis caused by hydrogen peroxide through the degradation of the transcription factor FOXO3a [45]. SIRT1 also has an anti-apoptotic effect by inactivating p53 via deacetylation, decreasing p53 transcriptional activity, and preventing p53-dependent apoptosis [46].

## 5. Conclusion

The current study demonstrated that fisetin ameliorates MSG-induced testicular toxicity via both central and peripheral mechanisms. It acts centrally by stimulating the hypothalamic-pituitary-gonadal axis and increasing plasma sex hormone levels. It also has a peripheral effect on the testis, activating the testicular SIRT1/ pAMPK signaling pathway and inhibiting glutamate-induced oxidative stress. The protective effects of fisetin against MSG-induced testicular toxicity and other causes of male infertility should be investigated on a human scale.

## Author contributions

FHR designed the study, analyzed data, and interpreted the results. FHR, NAS, SEA, HAM, MTA, RAE, OAE, RAM, SSAM, and AAE performed experiments. All authors drafted the manuscript and approved its final version.

## Data Availability

All datasets generated or analyzed in the study are included in this article.
